# Weak X‐Ray to Visible Lights Detection Enabled by a 2D Multilayered Lead Iodide Perovskite with Iodine‐Substituted Spacer

**DOI:** 10.1002/advs.202301149

**Published:** 2023-05-10

**Authors:** Shihai You, Panpan Yu, Jianbo Wu, Zeng‐Kui Zhu, Qianwen Guan, Lina Li, Chengmin Ji, Xitao Liu, Junhua Luo

**Affiliations:** ^1^ State Key Laboratory of Structural Chemistry Fujian Institute of Research on the Structure of Matter Chinese Academy of Sciences Fuzhou Fujian 350002 P. R. China; ^2^ Fujian Science and Technology Innovation Laboratory for Optoelectronic Information of China Fuzhou Fujian 350108 P. R. China; ^3^ Key Laboratory of Fluorine and Silicon for Energy Materials and Chemistry of Ministry of Education School of Chemistry and Chemical Engineering Jiangxi Normal University Nanchang Jiangxi 330022 P. R. China; ^4^ University of Chinese Academy of Sciences Beijing 100049 P. R. China

**Keywords:** 2D hybrid perovskite, broadband photodetector, low detection limit, X‐ray detection

## Abstract

Broadband photodetectors (PDs) with low detection limits hold significant importance to next‐generation optoelectronic devices. However, simultaneously detecting broadband (i.e., X‐ray to visible regimes) and weak lights in a single semiconducting material remains highly challenging. Here, by alloying iodine‐substituted short‐chain cations into the 3D FAPbI_3_ (FA = formamidine), a new 2D bilayered lead iodide hybrid perovskite, (2IPA)_2_FAPb_2_I_7_ (**1**, 2IPA = 2‐iodopropylammonium), that enables addressing this challenge is reported. Such a 2D multilayered structure and lead iodide composition jointly endow **1** with a minimized dark current (6.04 pA), excellent electrical property, and narrow bandgap (2.03 eV), which further gives it great potential for detecting broadband weak lights. Consequently, its high‐quality single crystal PDs exhibit remarkable photoresponses to weak ultraviolet–visible lights (377–637 nm) at several tens of nW cm^−2^ with high responsivities (>10^2^ mA W^−1^) and significant detectivities (>10^12^ Jones). Moreover, **1** has an excellent X‐ray detection performance with a high sensitivity of 438 µC Gy^−1^ cm^−2^ and an ultralow detection limit of 20 nGy s^−1^. These exceptional attributes make **1** a promising material for broadband weak lights detection, which also sheds light on future explorations of high‐performance PDs based on 2D hybrid perovskites.

## Graphical abstract

A single‐crystal photodetector based on a novel 2D bilayered lead iodide perovskite with iodine‐substituted spacer, (2‐iodopropylammonium)_2_(formamidine)Pb_2_I_7_, is successfully developed for simultaneously detecting broadband (X‐ray to visible regimes) and ultraweak (nW cm^−2^ or nGy s^−1^ scale) lights with excellent performance, which sheds light on future explorations of 2D hybrid perovskites for next‐generation optoelectronic applications.

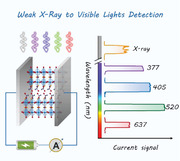

## 1 Introduction

Photodetectors (PDs), which convert optical signals into electrical signals, have deeply permeated into today's electronic industry as well as our daily life.^[^ ^1^ ^]^ Among them, broadband PDs that enable detecting a wide range of electromagnetic waves (i.e., X‐ray, ultraviolet (UV), visible (vis), and infrared (IR) lights) are especially attractive, owing to their importance to various applications, such as consumer electronics, optical communication, environmental monitoring, multispectral imagery, medical diagnosis, and so on.^[^ ^2^ ^]^ Conventionally, broadband PDs are fabricated by combining various photosensitive semiconductors, for instance, *α*‐Se for X‐ray, GaN for UV, Si for vis to near IR, and InGaAs for IR, which do achieve broad‐spectrum detection function. However, such a combination not only raises the volume and cost of optoelectronic systems but also increases operational complexity and data instability.^[^ ^2a^ ^]^ Meanwhile, the sensitive detection of weak lights has also gained growing interest in the fields of quantum communication, night/underwater surveillance, military, biomedical imaging, diagnostic radiology, etc.^[^ ^3^ ^]^ For example, the effective detection of weak X‐rays is highly desirable in medical imaging to reduce the risk of human body exposure to large X‐ray dosage.^[^ ^4^ ^]^ In these regards, there is an urgent need to develop photodetection materials capable of responding to multispectral weak lights. Although a large variety of semiconducting materials, including Si, group III‐V and II‐VI compounds, transition‐metal dichalcogenides, graphene, and black phosphorus, have been exploited, and significant progress has been made,^[^ ^2^, ^5^ ^]^ these materials still confront the tough challenge of simultaneous realization of broadband (i.e., X‐ray, UV, and vis regimes) and weak (i.e., nW cm^−2^ scale) lights response. Du et al. fabricated a fluorographene/graphene heterostructure PD, which shows broadband photoresponse from UV to mid‐IR wavelengths; but it fails to obtain a low detection limit restricted by its high dark current (µA scale).^[^ ^2b^ ^]^ Additionally, these inorganic semiconducting materials are limited in their practical applications due to the high cost and complexity of fabrication approaches, such as chemical vapor deposition and molecular beam epitaxy.^[^ ^1a^ ^]^ Therefore, exploring new photosensitive semiconducting materials capable of responding to broadband weak lights as well as with easy and inexpensive fabrication is of great significance for developing the next‐generation high‐performance PDs.

In recent years, organic–inorganic hybrid perovskites (OIHPs) have emerged as a class of appealing photodetection materials owing to their solution‐processability and impressive optical and electrical properties, such as large light absorption coefficient, low excitons binding energy, high charge mobility, and tunable bandgaps.^[^ ^1^, ^5^, ^6^ ^]^ For example, the MAPbI_3_ (MA = methylammonium) represented 3D OIHPs have been widely utilized as broadband and radiation PDs, however, the large dark current and severe ion migration have limited their ability to detect weak optical signals.^[^ ^7^ ^]^ Moreover, they suffer from serious environmental and operational instability. In contrast, 2D layered OIHPs, adopting a structure of alternative inorganic slabs and organic spacer layers, not only offer intrawell channels for carrier transport but also provide resistive organic barriers to suppress ion migration, minimize dark current, and improve stability, thus showing great promise for weak lights detection.^[^ ^6^, ^8^ ^]^ Liang et al. successfully realized the weak UV light (377 nm, ≈80 nW cm^−2^) detection using a 2D (BA)_2_PbBr_4_ (BA = *n*‐butylamine) single crystal (SC) PD; but limited by its wide bandgap, this device cannot respond to a broad range of electromagnetic waves.^[^ ^8d^ ^]^ 2D lead iodide multilayered OIHPs typically have a relatively narrow bandgap as well as an excellent optoelectronic performance, which enable them with broadband light absorption (i.e., from UV to NIR), thus holding great potential as broadband PDs with low detection limits.^[^ ^9^ ^]^ Han et al. fabricated a SC PD based on (*i*PA)_2_EA_2_Pb_3_I_10_ (*i*PA = *iso*‐pentylammonium, EA = ethylammonium), which demonstrated significant responses to broadband optical signals from 365 to 670 nm; but its weak light detection performance was not explored.^[^ ^9a^ ^]^ Besides, the presence of high atomic number (*Z*) and heavy elements of Pb and I makes lead iodide OIHPs particularly suitable for X‐ray detection.^[^ ^7c^ ^]^ However, to our best knowledge, there is no report on the simultaneous detection of ultraweak X‐ray, UV, and vis lights using a sole OIHP. Inspired by above works, we are aiming to realize sensitive detection of weak X‐rays to UV–vis lights based on 2D lead iodide OIHPs.

In this work, we report a 2D bilayered lead iodide hybrid perovskite with iodine‐substituted short‐chain spacer, (2IPA)_2_FAPb_2_I_7_ (**1**, 2IPA = 2‐iodopropylammonium, FA = formamidinium), for broadband detection from X‐ray to vis lights with low detection limits. This lead iodide composition of **1** gives it a narrow bandgap (2.03 eV), while the 2D structure endows it with the suppressed ion migration and minimized dark current (6.04 pA at 10 V bias), which then synergistically enable it with the capability of detecting broadband weak light signals. As expected, PDs based on high‐quality SCs of **1** show remarkable photoresponses to weak UV–vis lights (377–637 nm) at a nW cm^−2^ scale. Specifically, a notable photocurrent on/off ratio (7.5 × 10^3^), a high responsibility (*R*) of 776 mA W^−1^, an excellent detectivity (D*) of 8.36 × 10^12^ Jones, a fast response time (≈300 µs), and an extremely low detection limit of 11.55 nW cm^−2^ were achieved under 520 nm illumination. Impressively, **1** also exhibits excellent X‐ray detection performance with a sensitivity of 438 µC Gy^−1^ cm^−2^ and an ultralow detection limit of 20 nGy s^−1^. These outstanding detection performances for broadband weak lights suggest that **1** is a promising candidate for next‐generation high‐performance PDs.

## 2 Results and Discussion

### 2.1 Crystal Structure

**Figure 1:**
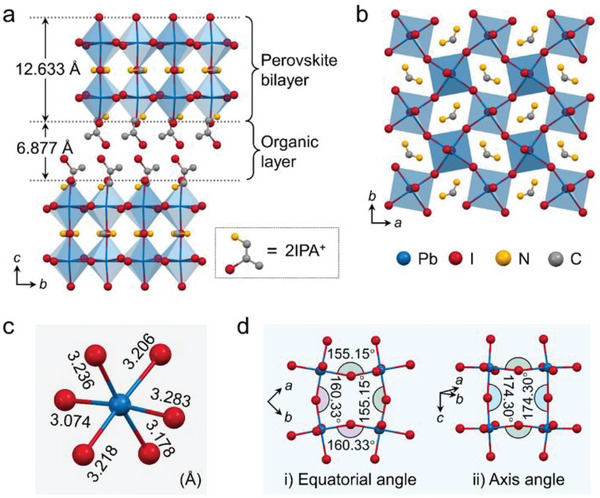
Crystal structure of **1**. a) The packing structure of **1** viewed along the *a*‐axis. b) Top‐down view of the inorganic layer, c) the [PbI_6_]^4−^ octahedron with labeled bond lengths, and d) the equatorial and axis angles of the inorganic layer in **1**. Hydrogen atoms are omitted for clarity.

The iodine‐substituted short‐chain spacer cation 2IPA was first synthesized through an iodination reaction of iso‐propanolamine (Figure S1, Supporting Information).^[^ ^10^ ^]^ Then, the dark red single crystal plates of **1** were grown through a slow temperature‐cooling process from the solution of concentrated hydroiodic acid containing stoichiometric amounts of raw materials, and its phase purity was identified by the powder X‐ray diffraction (XRD) as shown in Figure S2 (Supporting Information). Figure S3 (Supporting Information) demonstrates that this compound is environmentally stable and exhibits high thermal stability up to 212 °C. Single crystal XRD reveals that **1** crystallizes in the orthorhombic crystal system with the space group of *P* *b* *c* *m* (Table S1, Supporting Information). As shown in **Figure** **1**a, the general structure of **1** adopts a typical 2D Ruddlesden–Popper (RP)‐type motif, where the inorganic [Pb_2_I_7_]^∞^ perovskite bilayers composed of corner‐sharing [PbI_6_]^4−^ octahedra and the organic 2IPA layers are alternated and stacked along the *c*‐axis. Through the interaction of N—H∙∙∙I hydrogen bonds, 2IPA cations are firmly anchored in the interlayer space between two inorganic perovskite bilayers, while small FA cations are confined in the cages enclosed by corner‐shared [PbI_6_]^4−^ octahedra within the inorganic slabs (Figure S4, Supporting Information). The introduction of high *Z* and heavy element of I into spacer cation leads to a larger density (3.69 g cm^−3^) of **1** than those of 2D OIHPs with nonhalogenated spacers, like (BA)_2_FAPb_2_I_7_ (3.12 g cm^−3^),^[^ ^11^ ^]^ (*i*BA)_2_MAPb_2_I_7_ (3.26 g cm^−3^, *i*BA = *iso*‐butylamine),^[^ ^12^ ^]^ (PA)_2_FAPb_2_I_7_ (3.03 g cm^−3^, PA = *n*‐pentylamine),^[^ ^13^ ^]^ consequently favoring stronger high‐energy X‐ray absorption. The inorganic slab thickness is measured to be 12.633 Å. It is comparable to those of reported 2D bilayered lead iodide OIHPs but much larger than those of 2D monolayered lead iodide compounds (≈6.3 Å), which could provide more efficient carrier transport channels. The interlayer distance of **1** is determined to be 6.877 Å, and it is slightly larger than those of some lead iodide perovskites with similar short‐chain spacers, including (*i*BA)_2_MAPb_2_I_7_ (6.568 Å) and (*i*BA)_2_GPb_2_I_7_ (6.468 Å, G = guanidine),^[^ ^12^, ^14^ ^]^ due to the bulkiness and steric interaction of the C—I bond (≈2 Å). But it is shorter than the interlayer distance of those OIHPs with long‐chain spacers, such as (BA)_2_FAPb_2_I_7_ (7.032 Å) and (PA)_2_FAPb_2_I_7_ (7.938 Å),^[^ ^11^, ^13^ ^]^ which could benefit to better carrier transport. As presented in Figure 1b, the inorganic perovskite slab is isotropic along the *a*‐ and *b*‐axis. Moreover, the two individual [PbI_4_]^∞^ layers overlap exactly on the top/bottom of each other without any displacement. Such an offset‐free stack behavior is different from that of many reported 2D bilayered lead iodide perovskites with obvious displacement between two [PbI_4_]^∞^ layers (Figure S5, Supporting Information), such as (*i*BA)_2_MAPb_2_I_7_,^[^ ^12^ ^]^ (*i*BA)_2_GPb_2_I_7_,^[^ ^14^ ^]^ (HA)_2_GPb_2_I_7_ (HA = hexylamine),^[^ ^15^ ^]^ and (PEA)_2_MAPb_2_I_7_ (PEA = phenylethylamine),^[^ ^16^ ^]^ which suggests **1** is of less distortion and better electrical performance.

Furtherly, the distortion level of the basic building unit [PbI_6_]^4−^ octahedron is evaluated by calculating the bond length distortion (Δ*d*)^[^ ^17^ ^]^

(1) $$\Delta d=\frac{1}{6}\sum_{i=1}^{6}{\left(\frac{d_{i}-d}{d}\right)}^{2}$$

where *d* and *d*_*i*_ are the mean and individual Pb—I bond lengths. As labeled in Figure 1c, the Pb—I bond lengths range from 3.074 to 3.283 Å, which gives a Δ*d* of 4.05 × 10^−4^. This value is slightly smaller than those of some similar 2D lead iodide OIHPs, such as (*i*BA)_2_GPb_2_I_7_ (5.80 × 10^−4^) and (PA)_2_FAPb_2_I_7_ (6.20×10^−4^).^[^ ^13^, ^14^ ^]^ To further quantify the distortion across the inorganic layer, the equatorial (along the inorganic layer) and axis (perpendicular to the inorganic layer) I‐Pb‐I angles were measured (Figure 1d).^[^ ^18^ ^]^ The axis I‐Pb‐I angle is determined to be 174.30°, which is close to the standard 180° and comparable to those of many reported RP‐type 2D bilayered lead halide OIHPs. The average equatorial angle is measured to be 157.74°, and it is a little bigger than those of (*i*BA)_2_MAPb_2_I_7_ (157.09°), (PEA)_2_MAPb_2_I_7_ (153.87°),^[^ ^16^ ^]^ and (PMA)_2_CsPb_2_Br_7_ (152.55°, PMA = phenylmethylamine).^[^ ^19^ ^]^ This larger average value of equatorial angles implies that **1** has a relatively flatter inorganic layer, which is good for carrier transport.^[^ ^20^ ^]^ Overall, according to the crystal structure analysis, **1** has a flat and thick inorganic slab, composed of less distorted [PbI_6_]^4−^ octahedra, without displacement as well as with relatively short interlayer distance, which is advantageous for better charge carriers transport and thus favors better electrical properties.

Figure S6a,b (Supporting Information) shows the scanning electron microscope (SEM) images for **1**, in which the microcrystal demonstrates a very smooth surface without defects along (100) crystallographic plane, indicating the high crystal quality of **1**. Its energy dispersive X‐ray spectroscopy (EDS) elemental mappings (Figure S6c–f, Supporting Information) reveal that Pb, I, C, and N elements are homogeneously distributed within the crystal, suggesting the composition uniformity of **1**.

### 2.2 Semiconducting Properties

**Figure 2:**
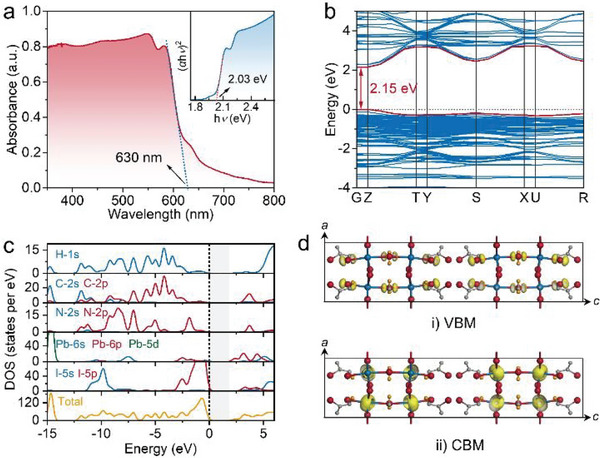
Band structure of **1**. a) The absorption spectrum and estimated optical bandgap (inset) of **1**. The calculated b) band structure, c) partial and total DOS profiles, d) charge density isosurfaces for the VBM and CBM of **1** using the DFT method.

The optical bandgap of **1** was measured using a UV–vis–NIR spectrophotometer. As shown in **Figure** **2**a, the optical absorption spectrum of **1** has an absorption edge at ≈630 nm, from which its optical bandgap is determined to be 2.03 eV by fitting the Tauc's formula.^[^ ^21^ ^]^ Such a narrow bandgap thus greatly favors the purpose of designing broadband PDs.^[^ ^9a^ ^]^ Furtherly, the density functional theory (DFT) calculations on electronic band structure and partial and total density of states (DOS) of **1** reveal that it features a direct bandgap with its valence band maximum (VBM) and conduction band minimum (CBM) locating at the same *G* point (Figure 2b). The calculated bandgap is 2.15 eV, comparable with the experimental value. Moreover, the partial DOS profiles indicate that the CBM mainly stems from the Pb‐6p orbitals, while the VBM is mainly determined by the I‐5p states (Figure 2c). Therefore, the optical bandgap and electronic structure of **1** are chiefly governed by the inorganic framework, which is further supported by its charge density distribution of VBM and CBM, as presented in Figure 2d. The bulk resistivity (*ρ*) of **1** SC is determined to be 3.84 × 10^10^ Ω cm (Figure S7, Supporting Information), which is much greater than the value of 10^7^–10^8^ Ω cm for 3D MAPbX_3_ (X = Cl, Br, I) perovskites.^[^ ^22^ ^]^ Such a large resistivity allows an extremely low dark current and noise current and thus contributes to the high‐performance detection of weak lights.^[^ ^8a^ ^]^

### 2.3 UV–Vis Lights Detection

**Figure 3:**
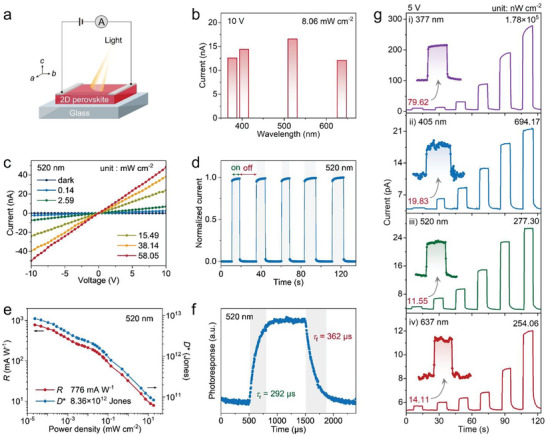
UV–vis lights detection performance of **1** SC PDs. a) Schematic illustration of the two‐terminal lateral PD based on **1** SC. b) The photocurrent response of **1** PD to 377, 405, 520, and 637 nm lights with a same laser power density. c) *I*–*V* traces of **1** detector measured in the dark and under 520 nm light illumination with varied intensities. d) Temporal photocurrent response of the **1** device under 520 nm light illumination and 5 V bias. e) *R* and D* of **1** PD under 520 nm illumination calculated from the photocurrent measured at a 10 V bias. f) The response time of **1** device during the illumination on/off switching. g) *I*–*t* curves of **1** PD under weak light illumination of different wavelengths (i.e., 377, 405, 520, and 637 nm).

Owing to the impressive semiconducting properties of **1**, we further evaluated its photodetection performance for a broadband spectrum ranging from UV to green and red lights. Two‐terminal lateral PDs, Ag/**1** SC/Ag, based on high‐quality single crystals of **1** were fabricated (**Figure** **3**a; and Figure S8, Supporting Information). As shown in Figure 3b; and Figure S9 (Supporting Information), **1** exhibits significant photoresponses across a wide wavelength range from 377 to 637 nm, which is consistent with its narrow bandgap. Under a same light power density of 8.06 mW cm^−2^, the maximum current under illumination (*I*_light_) is obtained at 520 nm, therefore it was chosen as the representative light for the further evaluation of photodetection performance. Figure 3c shows the current–voltage (*I*–*V*) traces of **1** device in the dark and under 520 nm light illumination. Their symmetric and linear behaviors indicate a good Ohmic contact with a small contact resistance between single crystal and Ag electrodes.^[^ ^13^ ^]^ Notably, this PD presents an extremely low dark current (*I*_d_) of 6.04 pA at a 10 V bias, which is much lower than those of many previously reported 2D bilayered lead iodide perovskite SC detectors, i.e., (*i*BA)_2_MAPb_2_I_7_ (49 pA at 10 V),^[^ ^12^ ^]^ (*i*BA)_2_GPb_2_I_7_ (46 pA at 10 V),^[^ ^14^ ^]^ and (3AMPY)FAPb_2_I_7_ (83 pA at 10 V, 3AMPY = 3‐(aminomethyl)piperidinium).^[^ ^9c^ ^]^ Such a low *I*_d_ suggests the low intrinsic carrier concentration and high quality of **1** SCs, which is beneficial to achieving high‐performance photodetection. Under illumination, the current rises sharply due to the photoelectric effect induced higher carrier concentration. Specifically, *I*_light_ increases to 48.28 nA under a light intensity of 58.05 mW cm^−2^ (10 V bias). This quite low *I*_d_ and considerable *I*_light_ thus produces a large on/off ratio of 7.5×10^3^, revealing the great potential of **1** as an efficient photodetector material. Figure 3d presents the steady photoresponse of **1** PD after long‐time light on/off switching cycles. Further, two critical parameters, *R* and D*, are introduced to assess the performance of this photodetector (detailed calculation see the Supporting Information).^[^ ^8a^ ^]^ Generally, *R* reflects the sensitivity of a photodetector to incident lights, while D* stands for the ability of a device for detecting weak light signals.^[^ ^23^ ^]^ As shown in Figure 3e, *R* and D* show a gradual increase with the decreasing light power densities, and their maximum values are determined to be 776 mA W^−1^ and 8.36 × 10^12^ Jones (at 10 V bias and 23.1 nW cm^−2^), respectively. These values are superior to those of recently reported 2D hybrid perovskites SC PDs, i.e., (BA)_2_CsPb_2_Br_7_ (39.5 mA W^−1^ and 1.2 × 10^12^ Jones at 0 V and 40 nW cm^−2^),^[^ ^8e^ ^]^ (BA)_2_PbBr_4_ (16.9 mA W^−1^ and 2.06 × 10^12^ Jones at 10 V and 80 nW cm^−2^),^[^ ^8d^ ^]^ and (3AMPY)FAPb_2_I_7_ (230 mA W^−1^ and 6 × 10^12^ Jones at 8 µW cm^−2^).^[^ ^9c^ ^]^ Impressively, not only for 520 nm light, **1** device also exhibits large on/off ratios (>10^3^), high *R* (>10^2^ mA W^−1^), and D* (>10^12^ Jones) for 377, 405, and 637 nm lights (Figures S10–S12 and Table S2, Supporting Information). These superior attributes reveal that **1** is a high‐performance broadband PD. The response time (*τ*) of **1** SC PD is demonstrated in Figure 3f, showing a fast response speed with a rise/fall time (*τ*_r_/*τ*_f_) of 292/362 µs, which is comparable to other reported 2D hybrid perovskite PDs.^[^ ^8^, ^12^ ^]^ Additionally, the photocurrent signals show no significant attenuation after about 10^3^ switching cycles (Figure S13, Supporting Information), suggesting the remarkable stability and high reliability of **1** SC PDs.

Figure S14 (Supporting Information) shows the correlation of photocurrents (*I*_ph_ = *I*_light_ – *I*_d_) with incident light power densities (*P*). It can be fitted by the power law equation *I*_ph_∝*P^*β*^*, where *β* represents the response of detector to the light intensity, reflecting the processes of electron–hole generation, trapping, and recombination.^[^ ^8^, ^24^ ^]^ Through a sublinear fit, the values for *β* are respectively estimated to be 0.76 and 0.52 under weak and strong 520 nm light illumination. Such a decrease of *β* can be ascribed to the intensified recombination loss of carriers under intense light illumination due to the higher photogenerated carrier concentration as well as the presence of some trap states between Fermi level and the conduction band.^[^ ^25^ ^]^ Here, the larger *β* value for small light power densities implies that **1** has a relatively low recombination activity in weak lights region and therefore is potential for weak lights detection. Thus, we further studied its detection performance for weak light signals. Figure 3g shows the current–time (*I*–*t*) curves of **1** SC device for weak lights of different wavelengths (i.e., 377, 405, 520, and 637 nm). It is clear that **1** PD shows significant photoresponses to these lights at a nW cm^−2^ scale. Specifically, the detection limits for 377, 405, 520, and 637 nm lights are determined to be 79.62, 19.83, 11.55, and 14.11 nW cm^−2^, respectively, which are superior to its perovskite counterparts (Table S3, Supporting Information), such as (BA)_2_PbBr_4_ (80 nW cm^−2^ at 377 nm),^[^ ^8d^ ^]^ (BA)_2_CsPb_2_Br_7_ (40nW cm^−2^ at 405 nm),^[^ ^8e^ ^]^ and (FPEA)_2_MAPb_2_I_7_ (100 nW cm^−2^ at 520 nm, FPEA = 4‐fluorophenethylammonium).^[^ ^8f^ ^]^ Based on these results above, we concluded that **1** is a highly potential semiconducting material for detecting ultraweak UV–vis lights.

### 2.4 X‐Ray Detection

**Figure 4:**
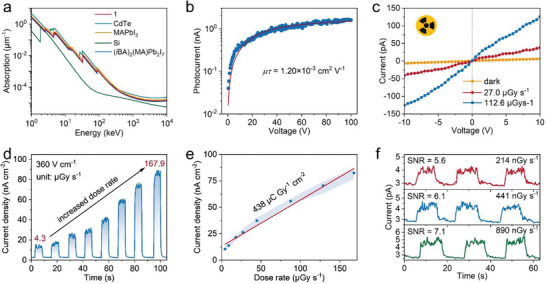
X‐ray detection performance of **1** SC detector. a) Absorption coefficients of **1**, CdTe, Si, and MAPbI_3_, (*i*BA)_2_MAPb_2_I_7_ as a function of X‐ray photon energy. b) The bias dependent photoconductivity of **1** detector under X‐ray irradiation. The inset is derived *µτ* value. c) *I*–*V* traces of **1** device in dark and under X‐ray irradiation. d) Photocurrent response of **1** detector to X‐ray with increased dose rates under an electric field of 360 V cm^−1^. e) X‐ray photocurrents as a function of dose rate. The inset is the sensitivity calculated by linearly fitting these data. f) *I*–*t* curves of **1** SC device under X‐ray irradiation with low dose rates. SNR values are also calculated.

Considering the excellent detection performance of **1** for UV–vis lights as well as the presence of high *Z* and heavy elements (i.e., Pb and I), we further explored its X‐ray detection performance. First, the absorption spectra over a broad range of photon energies (1 keV to 10 MeV) for **1** and some typical semiconductors for X‐ray detection (i.e., Si, CdTe, and MAPbI_3_) were simulated by using the photon cross‐section database.^[^ ^26^ ^]^ As plotted in **Figure** **4**a, the linear absorption coefficient of **1** across the entire energy region is much higher than that of Si and close to the values for CdTe and MAPbI_3_, revealing its excellent X‐ray absorption capability. When compared to a highly similar compound (*i*BA)_2_MAPb_2_I_7_,^[^ ^12^ ^]^ **1** also shows a higher absorption coefficient spanning the whole energy range (i.e., ≈20% at 50 keV), which is caused by the introduction of heavy I atoms into spacer cations. Figure S15 (Supporting Information) presents the attenuation efficiency of these materials for 50 keV hard X‐ray photons, which is the maximum energy of our X‐ray tube used to evaluate detection performance. Generally, a high attenuation efficiency allows a reduced crystal thickness required to completely absorb X‐ray photons.^[^ ^27^ ^]^ Specifically, at a thickness of 1 mm, **1** can attenuate 97.62% of the incident X‐ray photons, which approaches the ratio for the state‐of‐the‐art CdTe (99.80%) and is much higher than that of Si (9.71%). Efficient charge collection is critical for a high‐performance X‐ray detector, which can be assessed by the mobility‐lifetime (*µτ*) product.^[^ ^7^, ^27^, ^28^ ^]^ Here, we derived the *µτ* product by fitting the photoconductivity based on the simplified Hecht equation^[^ ^7c^ ^]^

(2) $$I=\frac{I_{0}\mu \tau V}{L^{2}}\left[1-\exp\left(-\frac{L^{2}}{\mu \tau V}\right)\right]$$

where *I* is the photocurrent, *I*_0_ is the saturated photocurrent, *L* is the distance between electrodes, *V* is the applied bias, *µ* is carrier mobility, and *τ* is carrier lifetime. As fitted in Figure 4b, the SC of **1** has a product of 1.20 × 10^−3^ cm^2^ V^−1^. It is comparable with those of some reported 2D hybrid perovskite SCs, such as (CH_3_OC_3_H_9_N)_2_CsPb_2_Br_7_ (3.2 × 10^−3^ cm^2^ V^−1^),^[^ ^29^ ^]^ (o‐FPEA)_2_PbI_4_ (3.79 × 10^−3^ cm^2^ V^−1^),^[^ ^30^ ^]^ and (3AMPY)FAPb_2_I_7_ (2.0 × 10^−3^ cm^2^ V^−1^),^[^ ^9c^ ^]^ but much larger than those of commercial *α*‐Se film (≈10^−7^ cm^2^ V^−1^) and MAPbI_3_ polycrystalline film (2 × 10^−7^ cm^2^ V^−1^).^[^ ^31^ ^]^ This relatively large *µτ* product coupling with the high bulk resistivity usually leads to a large device signal‐to‐noise ratio (SNR), thus endowing **1** with the capability of resolving X‐rays of low dose rates.

Owing to these merits including the high resistivity and low dark current, excellent X‐ray attenuation ability, high *µτ* product, and excellent semiconducting properties, **1** holds great promise for X‐ray detection. Here, a planar SC detector with Ag as the electrodes, Ag/**1** SC/Ag, was adopted (Figure S16, Supporting Information). Its detection performance was measured under irradiation of a silver target X‐ray tube with X‐ray photon energy up to 50 keV and peak intensity at 22 keV. Figure 4c shows the *I*–*V* traces of **1** SC detector in the dark and under X‐ray irradiation, which indicates an obvious photoresponse. The current for positive and negative voltages is symmetric, suggesting the suppressed ion migration in **1** SC.^[^ ^30^ ^]^ The *I*–*t* curves of this detector under X‐ray irradiation with increased dose rates are demonstrated in Figure 4d. Obviously, a stable dark current under a 360 V cm^−1^ electric field was obtained (at around 2.70 nA cm^−2^), which is different from the serious drift of the dark current in its 3D perovskite counterparts, further revealing the suppressed ion migration in 2D hybrid perovskites.^[^ ^9c^ ^]^ The photocurrent density shows a remarkable increase with the gradually increased X‐ray dose rate from 4.3 to 167.9 µGy s^−1^, demonstrating the excellent photoresponse of **1** to X‐ray irradiation. We also noticed that the photocurrent signals exhibited much larger fluctuations (noises) than the dark current, which mainly originates from the ripple in the output power of our X‐ray source, as well as possibly stems from the random electron–hole creation during the photoelectric process and the fluctuation related to the gain process.^[^ ^27^ ^]^

The sensitivity (*S*) and detection limit are two key parameters for evaluating the performance of an X‐ray detector (detailed calculation see the Supporting Information).^[^ ^27^, ^32^ ^]^ By linearly fitting the correlation between the signal current density and the X‐ray dose rate (Figure 4e), a high sensitivity of 438 µC Gy^−1^ cm^−2^ was achieved at a 360 V cm^−1^ electric field. This value is comparable with those of some typical 2D hybrid perovskites for X‐ray detection (Table S3, Supporting Information), such as (BDA)_2_PbI_4_ (242 µC Gy^−1^ cm^−2^ at 3100 V cm^−1^, BDA = butanediamine),^[^ ^33^ ^]^ (BA)_2_PbI_4_ (148 µC Gy^−1^ cm^−2^ at 100 V cm^−1^),^[^ ^34^ ^]^ and (CH_3_OC_3_H_9_N)_2_CsPb_2_Br_7_ (410 µC Gy^−1^ cm^−2^ at zero bias),^[^ ^29^ ^]^ but is 22 times higher than that of the commercial *α*‐Se X‐ray detector (20 µC Gy^−1^ cm^−2^ at 100 000 V cm^−1^).^[^ ^31^, ^32^ ^]^ Further, we studied the detection limit for **1** SC X‐ray detector. The International Union of Pure and Applied Chemistry (IUPAC) defines the detection limit as the equivalent X‐ray dose rate to produce a signal three times greater than the noise level. Hence, we took the dose rate with an SNR of 3 as the detection limit at a given electric field.^[^ ^27^ ^]^ Figure 4f shows that a high SNR of 5.6 for **1** detector is obtained when exposed to a low X‐ray dose rate of 214 nGy s^−1^, demonstrating its outstanding detection performance for weak X‐rays. Further fitting the correlation between SNR and dose rates (Figure S17, Supporting Information), an ultralow detection limit of 20 nGy s^−1^ can be derived, which is highly desired for practical medical imaging to reduce the risk of exposure to large X‐ray dosages. This low detection limit is comparable with a recently reported (FPEA)_2_PbI_4_ 2D perovskite SC device (23 nGy s^−1^),^[^ ^35^ ^]^ and is 275 folds lower than that of the commercial *α*‐Se for medical diagnostics (5.5 µGy s^−1^).^[^ ^31^ ^]^ The realization of the low detection limit in our device is mainly due to the extremely low dark current and inhibited ion migration. The dark current drift (*I*_drift_) is another important parameter for assessing the operational stability of detectors, which can be determined by the following equation^[^ ^36^ ^]^

(3) $$I_{\mathrm{drift}}=\left(I_{t}-I_{0}\right)/\left(E\times S\times t\right)$$

where the *I*_*t*_ and *I*_0_ are, respectively, dark current at time *t* and 0, *E* represents the electric field, and *S* is the device area. As presented in Figure S18 (Supporting Information), *I*_drift_ of **1** device is calculated to be 7.76 × 10^−6^ nA cm^−1^ s^−1^ V^−1^, which reveals its excellent operational stability.

We also investigated the environmental and operational stability of our SC PDs. After exposure to ambient air for 60 days, no significant reduction in photocurrent of **1** detector (without any organic package) was observed (Figure S19a, Supporting Information), demonstrating its exceptional environmental stability induced by the incorporation of iodine‐substituted organic cations of 2IPA. This detector was further exposed to continuous X‐ray irradiation of 1.99 mGy s^−1^ under a 360 V cm^−1^ electric field to evaluate its radiation stability (Figure S19b, Supporting Information). Following a total X‐ray dosage of 1.29 Gy, there is no obvious change in photocurrent and dark current, revealing the high operational stability of **1**.

## 3 Conclusion

By inserting an iodine‐substituted short‐chain spacer into 3D FAPbI_3_, we constructed a novel 2D bilayered lead iodide hybrid perovskite, (2IPA)_2_FAPb_2_I_7_ (**1**), for broadband weak lights detection from X‐ray to visible regimes. Its high‐quality single crystal photodetectors show remarkable photoresponses to wide‐spectral weak lights from 377 to 637 nm at a nW cm^−2^ scale with a high responsivity (>10^2^ mA W^−1^) as well as a considerable detectivity (>10^12^ Jones). Furthermore, **1** also exhibits an outstanding X‐ray detection performance with a high sensitivity of 438 µC Gy^−1^ cm^−2^ and an ultralow detection limit of 20 nGy s^−1^. Such a capability of broadband weak light detection can be ascribed to the narrow bandgap and multilayered 2D motif of **1**. This work not only provides a promising candidate for high‐performance broadband PDs but also throws insights on the further design of 2D hybrid perovskites for high‐performance optoelectronic applications.

## Conflict of Interest

The authors declare no conflict of interest.

## Supporting information

Supporting Information

Supporting Information

## Data Availability

The data that support the findings of this study are available from the corresponding author upon reasonable request.
