# Supplementary material for: Weak X‐Ray to Visible Lights Detection Enabled by a 2D Multilayered Lead Iodide Perovskite with Iodine‐Substituted Spacer
Source: Adv Sci (Weinh). 2023 May 10;10(21):2301149. doi: 10.1002/advs.202301149 (PMC10375184; doi:10.1002/advs.202301149)
Supplement: Supplementary file 1 — Supporting Information [file ADVS-10-2301149-s002.pdf]

ADVANCED SCIENCE
Open Access

Supporting Information

for *Adv. Sci.*, DOI 10.1002/advs.202301149

Weak X-Ray to Visible Lights Detection Enabled by a 2D Multilayered Lead Iodide Perovskite with Iodine-Substituted Spacer

*Shihai You, Panpan Yu, Jianbo Wu, Zeng-Kui Zhu, Qianwen Guan, Lina Li, Chengmin Ji, Xitao Liu and Junhua Luo**

Supporting Information

Weak X-Ray to Visible Lights Detection Enabled by a 2D Multilayered Lead Iodide Perovskite with Iodine-Substituted Spacer

Shihai You,[1] *Panpan Yu,*[2] *Jianbo Wu,*[1,3] *Zeng-Kui Zhu,*[1] *Qianwen Guan,*[1,3] *Lina Li,*[1,3,4] *Chengmin Ji,*[1,3,4] *Xitao Liu,*[1,3,4] *Junhua Luo*[1,2,3,4]*

[1] State Key Laboratory of Structural Chemistry, Fujian Institute of Research on the Structure of Matter, Chinese Academy of Sciences, Fuzhou, Fujian 350002, China

[2] Key Laboratory of Fluorine and Silicon for Energy Materials and Chemistry of Ministry of Education, School of Chemistry and Chemical Engineering, Jiangxi Normal University, Nanchang, Jiangxi 330022, China

[3] University of Chinese Academy of Sciences, Beijing 100049, China

[4] Fujian Science and Technology Innovation Laboratory for Optoelectronic Information of China, Fuzhou, Fujian 350108, China

Email: jhluo@fjirsm.ac.cn

Experimental Section

Raw materials. All chemicals, lead acetate trihydrate ($Pb(Ac)_2{\cdot}3H_2O$, 99.5%, Aladdin), hydroiodic acid solution (HI, 48% in water, Aladdin), formamidine acetate ($CH_4N_2{\cdot}CH_3COOH$, 99%, Aladdin), iso-propanolamine (C_3H_9ON, 99%, Macklin), hypophosphorous acid (H_3PO_2, 50% in water, Aladdin), for the synthesis were bought and used without further purification.

Synthesis. *2-Iodopropylammonium iodide* (*2IPA·I*). The iso-propanolamine (20 mmol; 1.52 g) was added into a mixture of 10 mL HI and 0.5 mL H_3PO_2 by heating to boiling for 2 h under constant magnetic stirring to obtain a clear solution. Then, the solution mixture was transferred on a hotplate (130 °C) to fully evaporate the solvent. At last, a white solid salt is produced (4.69 g, yield ~ 75%). The nuclear magnetic resonance (NMR) spectra were used to confirm the full conversion of iso-propanolamine to 2-iodopropylammonium iodide (**Figure S1**).[1]

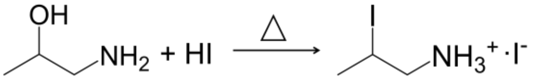

(*2IPA*)$_2$*FAPb*$_2$*I*$_7$. Crystalline materials of $(2IPA)_2FAPb_2I_7$ (**1**) were synthesized by dissolving $Pb(Ac)_2{\cdot}3H_2O$ (2 mmol; 758 mg), 2IPA·I (2 mmol; 616 mg), and $CH_4N_2{\cdot}CH_3COOH$ (FA, 1 mmol; 104 mg) powders in a mixture solution of 6 mL HI and 0.3 mL H_3PO_2 by heating to boiling under a constant magnetic stirring. After a clear yellow solution was obtained, the heating and stirring was stopped, and the solution was left on the hotplate to cool to room temperature. Then, small red crystals precipitated from the reaction solution. The bulk single crystals of **1** were grown from its saturated solution *via* a temperature-cooling process in an oven with a decrease rate of 1K day^{-1}.

Nuclear magnetic resonance (NMR) measurement. ^1H and ^{13}C NMR spectra of 2IPA cations were monitored using an Avance III 400 MHz spectrometer (Bruker, Germany). 50 mg 2IPA·I salt was dissolved in 1 mL DMSO-D6 for measurement.

Single crystal and powder X-ray diffraction (XRD). Single crystal XRD measurement of **1** was carried out on a D8 diffractometer with Mo Kα radiation (λ = 0.71073 Å) (Bruker, Germany). The crystal structure was solved by the direct method and refined by the full-matrix method based on F^2 using the SHELXTL program. Powder XRD patterns were measured on a Miniflex 600 X-ray diffractometer (Rigaku, Japan) in the 2-theta range of 5°- 40° with a step length of 0.02°.

Morphology characterization. The morphology and elemental distribution of a microcrystal of **1** were acquired using a JSM6700-F filed emission electron microscope (JEOL, Japan) attached with an X-Max^N energy dispersive X-ray spectroscope (Oxford Instruments, UK).

Thermogravimetric analysis. The thermogravimetric analysis for **1** powder was conducted on a STA 449 F3 Jupiter simultaneous thermal analyzer (Netzsch, Germany) under a nitrogen atmosphere from room temperature to 800°C with a rate of 15°C min^{-1}.

Optical property measurement. The diffuse reflection spectrum of **1** powders was measured on a Lambda 950 UV-Vis-NIR spectrometer (PerkinElmer, USA).

Ultraviolet-visible lights detection. The current-voltage (*I-V*) traces and current-time (*I-t*) curves were recorded using a high precision electrometer (Keithley 6517B, USA). Multiple-wavelength fiber-pigtailed laser diodes including 405, 520, and 637 nm (Thorlabs, USA) and a PiL037X 377 nm laser diode

(Advanced Laser Diode System (ALS), Germany) were used as light sources. The incident light intensity was measure by a PM100D optical power meter (Thorlabs, USA).

Calculations for responsivity (R) and detectivity (D*).[2] R can be calculated by the following euation,

$$R = I_{ph} / (P_i S) = (I_{light} - I_d) / (P_i S)$$

where I_{ph} is photocurrent, I_d and I_{light} represent the currents measured in the dark and under light illumination, respectively, P_i is the incident light intensity, and S is the effective illumination area. Assuming that the noise current is mainly contributed by the shot noise from dark current, D^* can be given by,

$$D^* = RS^{1/2}(2eI_d)^{-1/2}$$

in which e is the unit charge.

X-ray detection. The *I-V* traces and *I-t* curves under X-ray irradiation were also recorded using the 6517B high precision electrometer (Keithley, USA). A commercially available Ag target X-ray tube with X-ray photons energy up to 50 keV and peak intensity at 22 keV was used as the X-ray source (4 W, Mini-X2, Amptek, USA). The dose rate of X-ray tube was modulated by changing its tube current and measured by a commercial X-ray dosimeter (Accu-Gold, Radcal, USA) attached with the ion chamber (10X6-180 model) in an integrating mode.

Calculations of sensitivity (S) and signal-to-noise ratio (SNR).[3] S is defined as the collected charge per unit area under X-ray irradiation and can be determined by

$$S = (I_{x\text{-}ray} - I_d) / (D \times A)$$

where $I_{x\text{-}ray}$ and I_d are the currents recorded under X-ray irradiation and in the dark, respectively, D is the irradiation dose rate, and A is the effective area of detector.

SNR is calculated by,

$$SNR = I_{signal} / I_{noise}$$

where I_{signal} is the signal current and is derived by subtracting the average $I_{x\text{-}ray}$ ($\overline{I_{\text{x-ray}}}$) by the average dark current ($\overline{I_{\text{d}}}$), and I_{noise} is the noise current and is obtained by calculating the standard deviation of the $I_{x\text{-}ray}$.

$$I_{\text{signal}} = \overline{I_{\text{x-ray}}} - \overline{I_{\text{d}}}$$

$$I_{\text{noise}} = \sqrt{\frac{1}{n}\sum_{1}^{n}\left(I_i - \overline{I_{\text{x-ray}}}\right)^2}$$

Computation. Density functional theory (DFT) calculations were performed on the CASTEP program in the Materials Studio software. The exchange-correlation effects were treated using the Perdew-Burke-Ernzerhof function for solids (PBEsol) in the framework of generalized gradient approximation (GGA). A cutoff of 820 eV was used, and the *k*-space integration was carried out with a Monkhorst-Pack grid of 3×3×1. The norm-conserving pseudopotential was used to describe the interactions between the ionic cores and electrons. The orbital electrons of Pb ($5d^{10}6s^2 6p^2$), I ($5s^2 5p^5$), C ($2s^2 2p^2$), N ($2s^2 2p^3$), and H($1s^1$) were regraded as valence electrons.

Figures

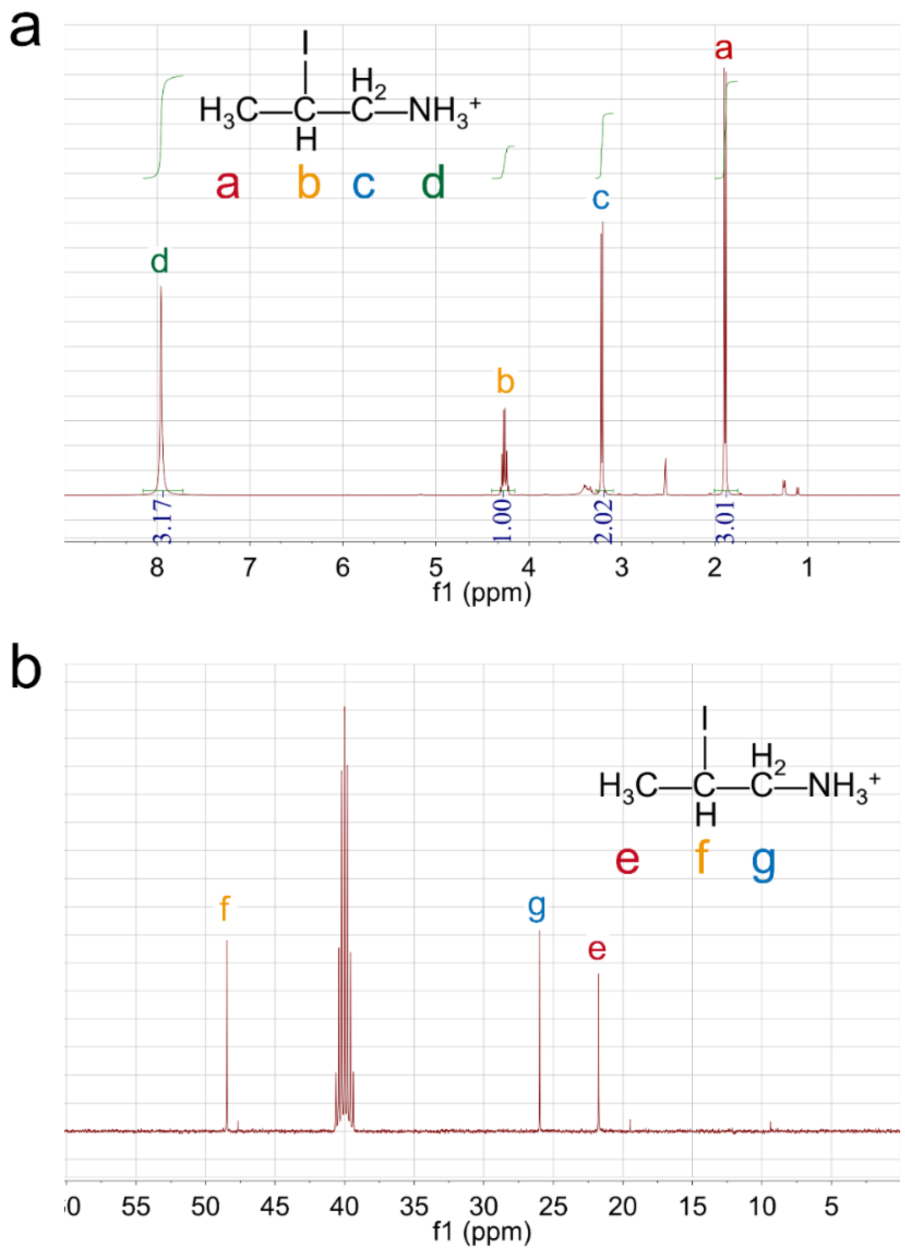

Figure S1. (a) ^{1}H and (b) ^{13}C NMR spectra of 2-iodopropylammonium (2IPA) cation.

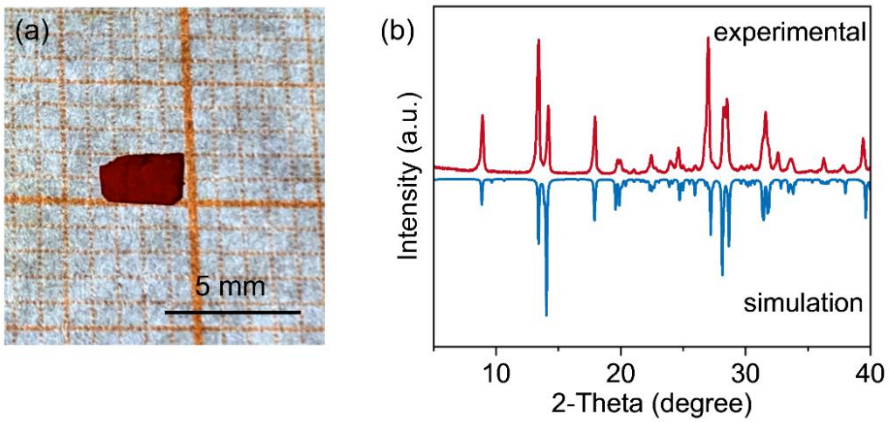

Figure S2. (a) Crystal photography, and (b) powder X-ray diffraction pattern for **1**.

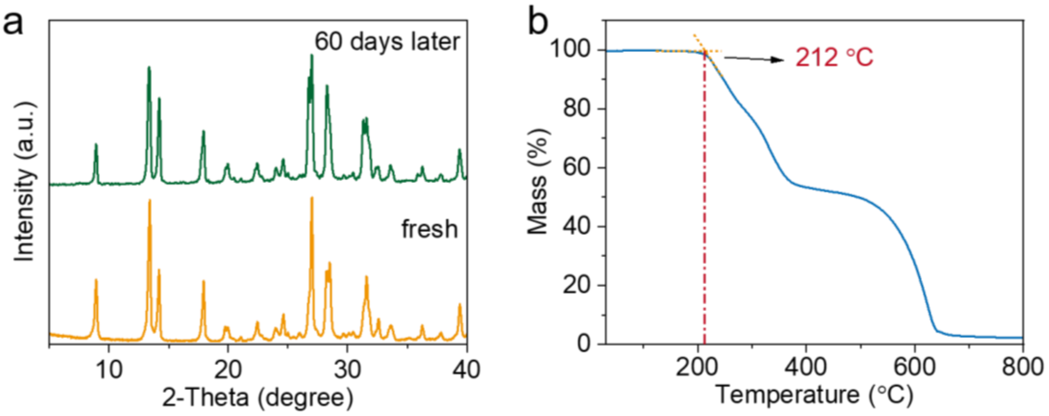

Figure S3. (a) PXRD patterns for **1** at fresh and after exposure to ambient air for one month. (b) TG curve of **1** under nitrogen atmosphere.

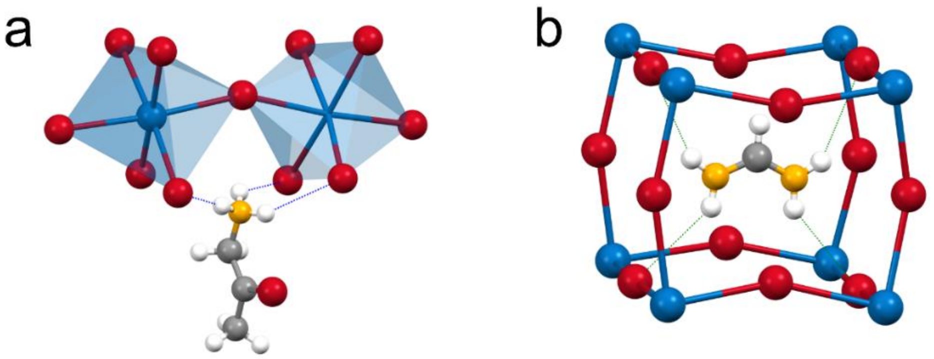

Figure S4. Hydrogen bonds for (a) 2IPA, and (b) FA cations in the crystal structure of **1**. (Red: I; Blue: Pb; Gray: C; Yellow: N; White: H.)

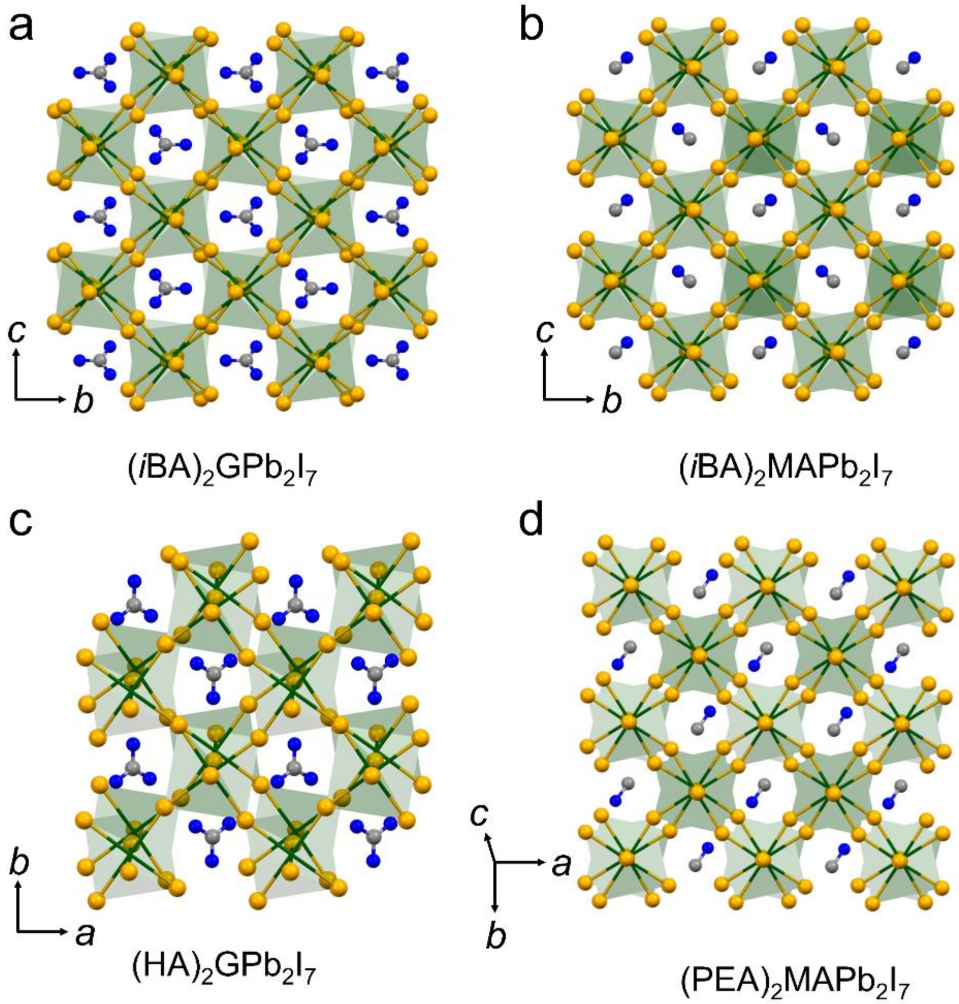

Figure S5. The perovskite bilayer of (a) $(i\text{BA})_2\text{GPb}_2\text{I}_7$ (*i*BA = iso-butylamine, G = guanidine),[4] (b) $(i\text{BA})_2\text{MAPb}_2\text{I}_7$ (MA = methylamine),[5] (c) $(\text{HA})_2\text{GPb}_2\text{I}_7$ (HA = hexylamine),[6] and (d) $(\text{PEA})_2\text{MAPb}_2\text{I}_7$ (PEA = phenylethylamine).[7] (Yellow: I; Green: Pb; Gray: C; Blue: N)

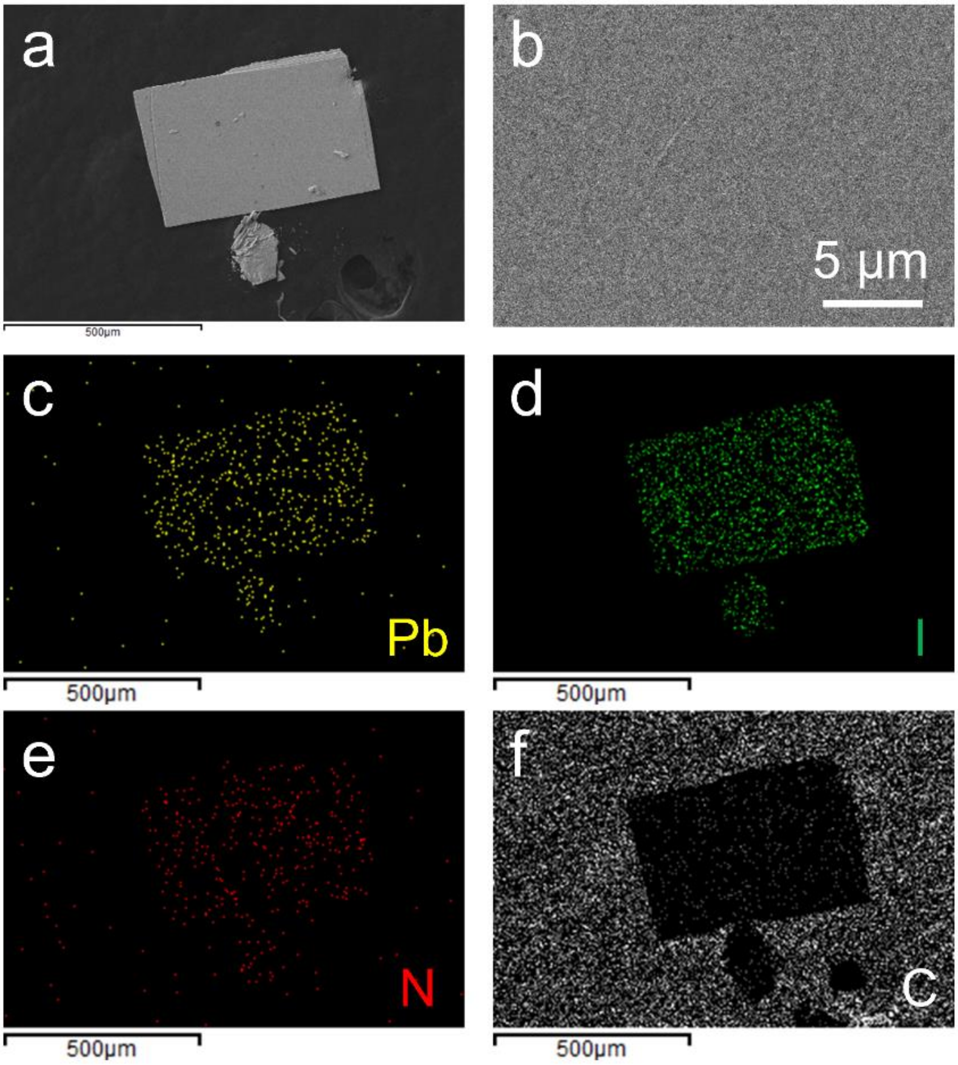

Figure S6. Scanning electron microscopy (SEM) images of (a) a selected crystal of **1**, and (b) an enlarged surface. (c-f) Energy-diffusive X-ray spectroscopy (EDS) mappings for Pb, I, N, and C.

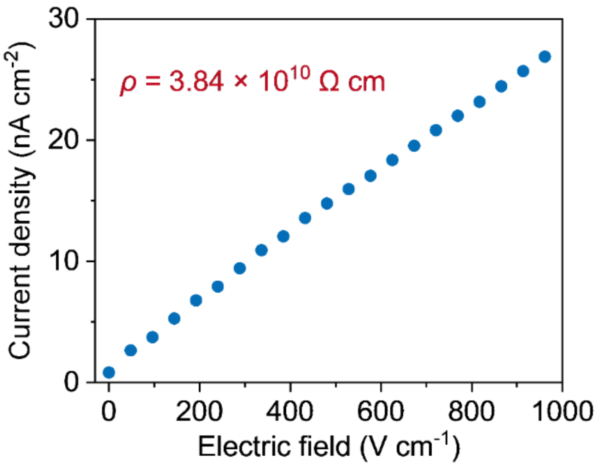

Figure S7. The bulk resistivity of **1** single crystal along the *ab* crystallographic plane.

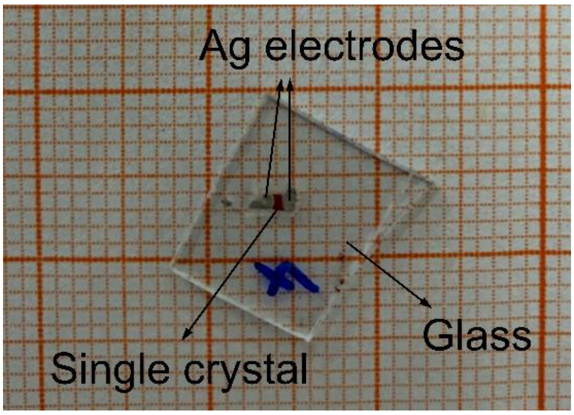

Figure S8. A photo for the planar device based on single crystal of **1**.

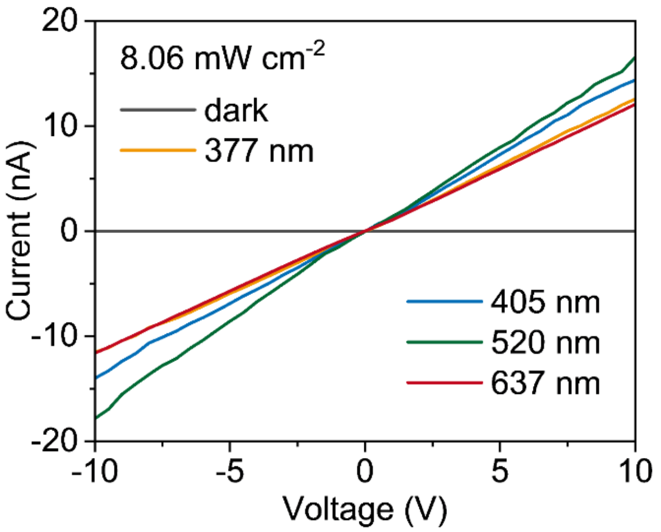

Figure S9. *I-V* traces of **1** single crystal photodetector measured under different wavelengths (*i.e.*, 377, 405, 520, and 637 nm) with a same power density of 8.06 mW cm^{-2}.

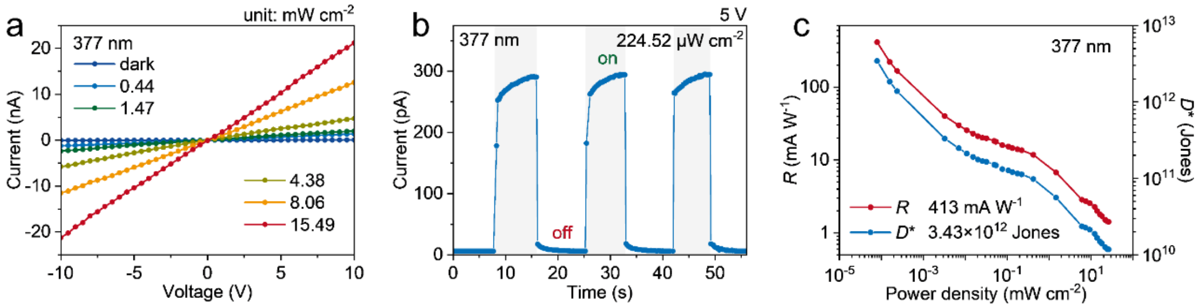

Figure S10. (a) *I-V* traces, (b) *I-t* curve, and (c) responsivity (*R*) and detectivity (*D**) of **1** single crystal photodetector under 377 nm light illumination.

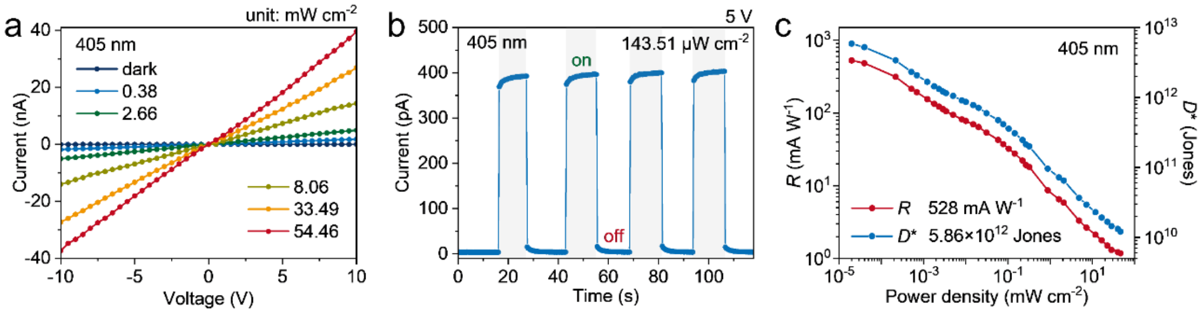

Figure S11. (a) *I*-*V* traces, (b) *I*-*t* curve, and (c) *R* and *D** of **1** single crystal photodetector under 405 nm light illumination.

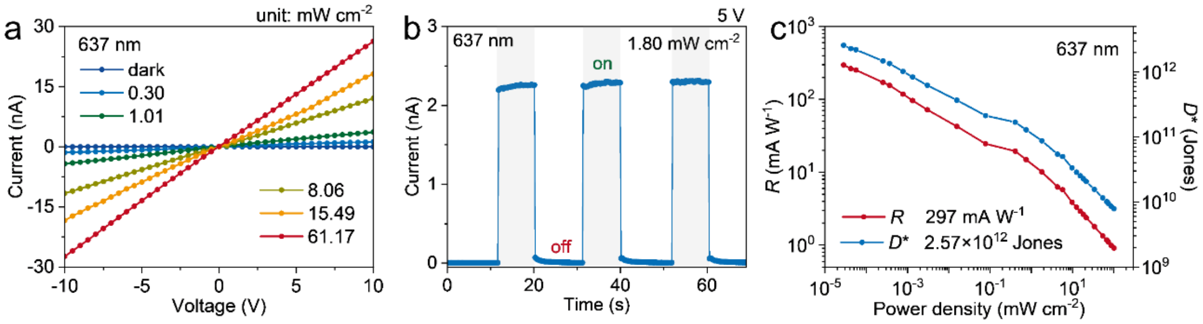

Figure S12. (a) *I*-*V* traces, (b) *I*-*t* curve, and (c) *R* and *D** of **1** single crystal photodetector under 637 nm light illumination.

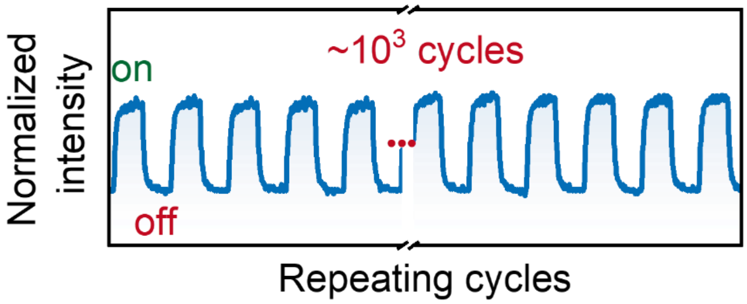

Figure S13. Repetitive switching cycles (~10^3) of photoresponse.

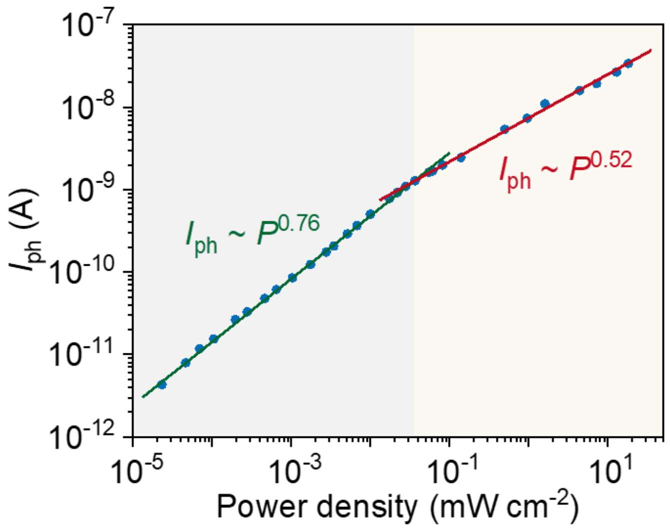

Figure S14. Power density dependent photocurrent of **1** under 520 nm illumination.

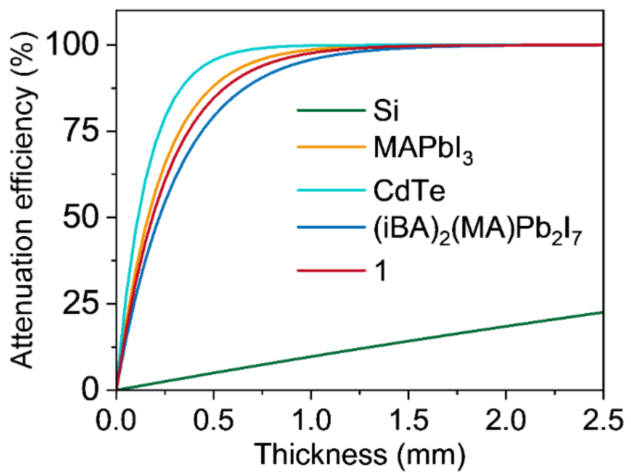

Figure S15. Attenuation efficiency of **1**, Si, CdTe, $MAPbI_3$ (MA = $CH_3NH_3^+$), and $(i\text{BA})_2(\text{MA})Pb_2I_7$ (*i*BA = iso-butylamine) for 50-keV X-ray photons versus thickness.

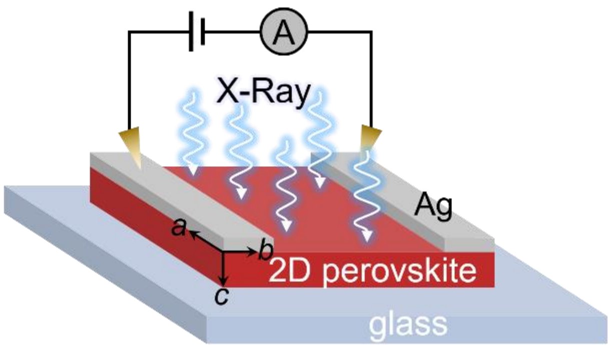

Figure S16. Schematical illustration of the two-terminal planar structured X-ray detector based on high quality single crystal of **1**.

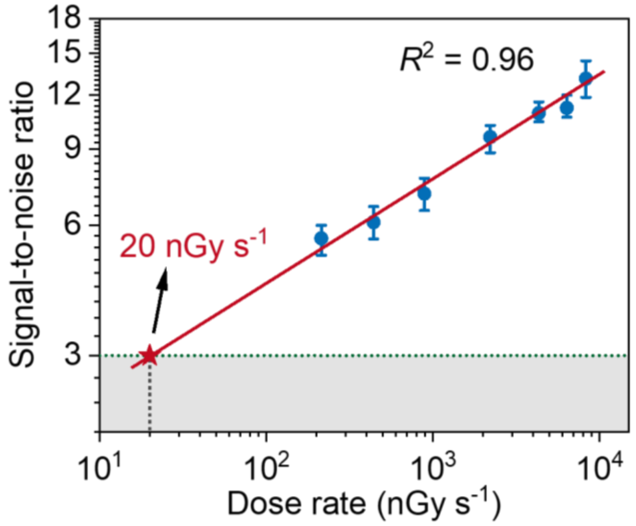

Figure S17. X-ray dose rate dependent signal-to-noise ratio (SNR) of the device under an electric field of 360 V cm^{-1}. The detection limit of 20 nGy s^{-1} is derived from the fitting line with an SNR of 3.

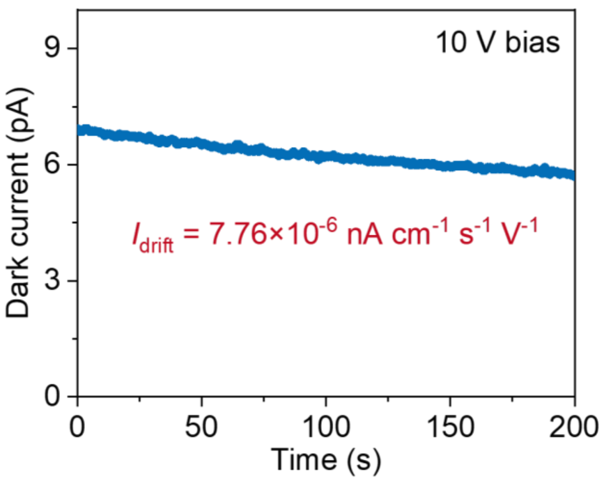

Figure S18. The dark cuurent drift (I_{drift}) of **1** SC device.

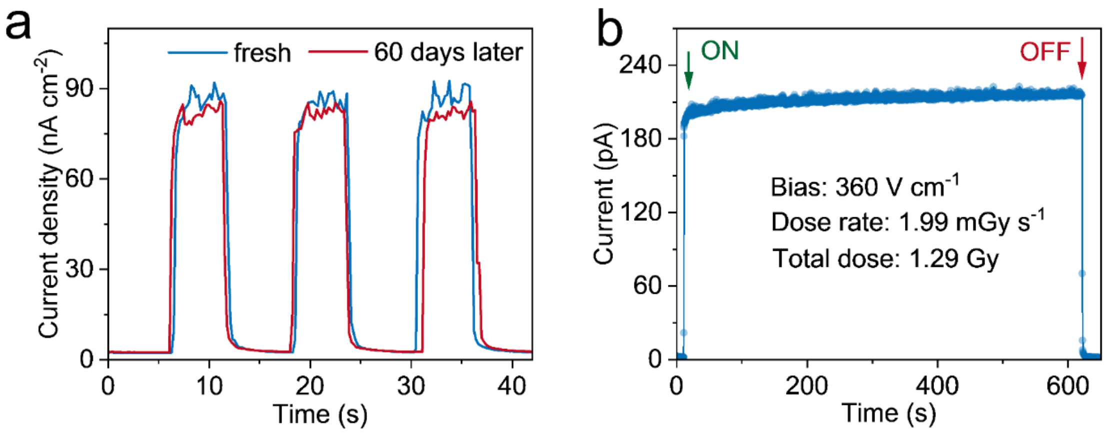

Figure S19. (a) I-t curves of 1 SC detector at fresh and after exposure to ambient air for 60 days. (b) Device operational stability resisting continuous X-ray irradiation under a high dose rate of 1.99 mGy s^{-1}.

Tables

Table S1. Crystal data and structure refinements for **1** at 150 K.

Formula	$(C_3H_9NI)_2(CH_5N_2)Pb_2I_7$
Temperature [K]	150
Weight	1719.79
Crystal system	Orthorhombic
Space group	*Pbcm*
a [Å]	8.9687(10)
b [Å]	8.8387(9)
c [Å]	39.021(4)
$\alpha = \beta = \gamma$ [°]	90
Volume [Å^3]	3093.2(6)
$\rho_{calc.}$ [g cm^{-3}]	3.693
Z	4
F(000)	2936.00
Radiation	MoKα (λ = 0.71073 Å)
2θ range for data collection [°]	4.524 ~ 55.04
Index ranges	$-11 \leq h \leq 11$, $-11 \leq k \leq 11$, $-50 \leq l \leq 50$
Reflections collected	46855
Independent reflections	3613 [R_{int} = 0.1134, R_{sigma} = 0.0449]
Final *R* indexes [$I \geq 2\sigma(I)$]	R_1 = 0.0437, wR_2 = 0.0835
Final *R* indexes [all data]	R_1 = 0.0728, wR_2 = 0.0963
GOF	1.052

Table S2. Key parameters for **1** SC PD under light illumination of different wavelengths (*i.e.*, 377, 405, 520, and 637 nm).

Wavelength (nm)	on/off ratio	*R* (mA W^{-1})	*D** (Jones)	Detection limit (nW cm^{-2})
377	3.3×10^3	413	3.43×10^{12}	79.62
405	6.2×10^3	528	5.86×10^{12}	19.83
520	7.5×10^3	776	8.36×10^{12}	11.55
637	4.5×10^3	297	2.57×10^{12}	14.11

Table S3. Photodetection performance comparison of **1** with some reported 2D hybrid perovskites for weak lights detection.

Compounds	Wavelength (nm)	on/off ratio	R (mA W^{-1})	D* (Jones)	τ_r/τ_f (μs)	Sensitivity (μC Gy^{-1} cm^{-2})	Detection limit (nW cm^{-2})	Ref.
**$(2IPA)_2FAPb_2I_7$**	X-Ray	/	/	/	292/362	438	20 nGy s^{-1}	This work
	377	3.3×10^3	413	3.43×10^{12}		/	79.62	
	405	6.2×10^3	528	5.86×10^{12}		/	19.83	
	520	7.5×10^3	776	8.36×10^{12}		/	11.55	
	637	4.5×10^3	297	2.57×10^{12}		/	14.11	
$(BA)_2PbBr_4$	377	~10^4	16.9	2.06×10^{12}	220/240	/	80	8
$(BA)_2FAPb_2Br_7$	405	/	0.5	1.47×10^{12}	220/370	/	82	9
$(BA)_2CsPb_2Br_7$	405	4.6×10^3	39.5	1.2×10^{12}	300/360	/	40	10
$(FPEA)_2MAPb_2I_7$ (FPEA= 4-fluorophenethylammonium)	520	9.0×10^5	114.7	5.10×10^{13}	273/339	/	100	11
$(BLA)_2CsAgBiBr_7$ /$Cs_2AgBiBr_6$ (BLA = benzylammonium)	520	~10^2	600	3.5×10^{14}	/	/	50	12
$(BA)_2PbI_4$	X-ray	/	/	/	/	148	241	13
$(BA)_2PbBr_4$	X-ray	/	/	/	/	726.18	8.20	14
$(FPEA)_2PbI_4$	X-ray	/	/	/	/	3402	23	15
$(BDA)_2PbI_4$ (BDA = butanediamine)	X-ray	/	/	/	/	242	430	16
$(CH_3OC_3H_9N)_2CsPb_2Br_7$	X-ray	/	/	/	/	410	/	17
$(o\text{-}F\text{-}PEA)_2PbI_4$ (FPEA= 2-fluorophenethylammonium)	X-ray	/	/	/	/	1724.5	460	18

References

[1] E. S. Vasileiadou, X. Jiang, M. Kepenekian, J. Even, M. C. De Siena, V. V. Klepov, D. Friedrich, I. Spanopoulos, Q. Tu, I. S. Tajuddin, E. A. Weiss, M. G. Kanatzidis, *J. Am. Chem. Soc.* **2022**, *144*, 6390-6409.

[2] D. Ji, T. Li, J. Liu, S. Amirjalayer, M. Zhong, Z.-Y. Zhang, X. Huang, Z. Wei, H. Dong, W. Hu, H. Fuchs, *Nat. Commun.* **2019**, *10*, 12.

[3] W. Pan, H. Wu, J. Luo, Z. Deng, C. Ge, C. Chen, X. Jiang, W.-J. Yin, G. Niu, L. Zhu, L. Yin, Y. Zhou, Q. Xie, X. Ke, M. Sui, J. Tang, *Nat. Photon.* **2017**, *11*, 726-732.

[4] X. Pan, H. Chen, L. Lu, S. Han, Y. Ma, J. Wang, W. Guo, H. Xu, J. Luo, Z. Sun, *Chem. Asian. J.* **2021,** *16*, 1925-1929.

[5] Y. Liu, Z. Wu, X. Liu, S. Han, Y. Li, T. Yang, Y. Ma, M. Hong, J. Luo, Z. Sun, *Adv. Opt. Mater.* **2019**, *7*, 1901049.

[6] Y. Fu, M. P. Hautzinger, Z. Luo, F. Wang, D. Pan, M. M. Aristov, I. A. Guzei, A. Pan, X. Zhu, S. Jin, *ACS Cent. Sci.* **2019,** *5*, 1377-1386.

[7] D. J. Morrow, M. P. Hautzinger, D. P. Lafayette, J. M. Scheeler, L. Dang, M. Leng, D. D. Kohler, A. M. Wheaton, Y. Fu, I. A. Guzei, J. Tang, S. Jin, J. C. Wright, *J. Phys. Chem. Lett.* **2020,** *11*, 6551-6559.

[8] L. Liang, X. Niu, X. Zhang, Z. Wang, J. Wu, J. Luo, *Adv. Opt. Mater.* **2022**, *10*, 2201342.

[9] Y. Ma, J. Wang, Y. Liu, S. Han, Y. Li, Z. Xu, W. Guo, J. Luo, M. Hong, Z. Sun, *J. Mater. Chem. C* **2021**, *9*, 881-887.

[10] J. Wang, Y. Liu, S. Han, Y. Ma, Y. Li, Z. Xu, J. Luo, M. Hong, Z. Sun, *Sci. Bull.* **2021,** *66*, 158-163.

[11] X. Hu, H. Xu, Y. Liu, L. Lu, W. Guo, S. Han, J. Luo, Z. Sun, *J. Phys. Chem. Lett.* **2022,** *13*, 6017-6023.

[12] X. Zhang, Y. Yao, L. Liang, X. Niu, J. Wu, J. Luo, *Angew. Chem. Int. Ed.* **2022**, *61*, e202205939.

[13] Yukta, J. Ghosh, M. A. Afroz, S. Alghamdi, P. J. Sellin, S. Satapathi, *ACS Photonics* **2022,** *9*, 3529-3539.

[14] X. Xu, Y. Wu, Y. Zhang, X. Li, F. Wang, X. Jiang, S. Wu, S. Wang, *Energ. Environ. Mater.* **2022**, e12478.

[15] H. Li, J. Song, W. Pan, D. Xu, W. A. Zhu, H. Wei, B. Yang, *Adv. Mater.* **2020**, *32*, e2003790.

[16] Y. Shen, Y. Liu, H. Ye, Y. Zheng, Q. Wei, Y. Xia, Y. Chen, K. Zhao, W. Huang, S. F. Liu, *Angew. Chem. Int. Ed.* **2020**, *59*, 14896-14902.

[17] C. Ji, Y. Li, X. Liu, Y. Wang, T. Zhu, Q. Chen, L. Li, S. Wang, J. Luo, *Angew. Chem. Int. Ed.* **2021**, *60*, 20970-20976.

[18] B. Zhang, Z. Xu, C. Ma, H. Li, Y. Liu, L. Gao, J. Zhang, J. You, S. Liu, *Adv. Funct. Mater.* **2022,** *32*, 2110392.
